# The Diabetic Cardiomyopathy: The Contributing Pathophysiological Mechanisms

**DOI:** 10.3389/fmed.2021.695792

**Published:** 2021-06-30

**Authors:** Teresa Salvatore, Pia Clara Pafundi, Raffaele Galiero, Gaetana Albanese, Anna Di Martino, Alfredo Caturano, Erica Vetrano, Luca Rinaldi, Ferdinando Carlo Sasso

**Affiliations:** ^1^Department of Precision Medicine, University of Campania Luigi Vanvitelli, Naples, Italy; ^2^Department of Advanced Medical and Surgical Sciences, University of Campania Luigi Vanvitelli, Naples, Italy

**Keywords:** diabetes mellitus, cardiomyopathy, heart failure, pathophysiology, insulin resistance

## Abstract

Individuals with diabetes mellitus (DM) disclose a higher incidence and a poorer prognosis of heart failure (HF) than non-diabetic people, even in the absence of other HF risk factors. The adverse impact of diabetes on HF likely reflects an underlying “diabetic cardiomyopathy” (DM–CMP), which may by exacerbated by left ventricular hypertrophy and coronary artery disease (CAD). The pathogenesis of DM-CMP has been a hot topic of research since its first description and is still under active investigation, as a complex interplay among multiple mechanisms may play a role at systemic, myocardial, and cellular/molecular levels. Among these, metabolic abnormalities such as lipotoxicity and glucotoxicity, mitochondrial damage and dysfunction, oxidative stress, abnormal calcium signaling, inflammation, epigenetic factors, and others. These disturbances predispose the diabetic heart to extracellular remodeling and hypertrophy, thus leading to left ventricular diastolic and systolic dysfunction. This Review aims to outline the major pathophysiological changes and the underlying mechanisms leading to myocardial remodeling and cardiac functional derangement in DM-CMP.

## Introduction

Diabetic Cardiomyopathy (DM-CMP) is a form of heart disease associated with diabetes mellitus (DM), which causes significant structural and functional changes in the myocardium. The pathogenesis has been a hot topic of research since its first description (1), and it is still under active investigation, as a complex interaction among multiple factors play a role at systemic, myocardial, and cellular/molecular levels. The current pathogenic hypotheses mostly derive from translational models, with human evidence far less developed due to limited access to human tissue samples.

This review aims to outline the state of the art about the major pathophysiological changes and underlying mechanisms leading to myocardial remodeling and cardiac functional derangement in DM-CPM.

## Diabetes Mellitus and Heart Failure: A Bidirectional Epidemiologic Association

The risk for heart failure (HF), as well as that for all components of cardiovascular disease (CVD), is higher in individuals with diabetes as compared to non-diabetic people.

The Framingham Heart Study, published in 1974, is among the first studies to demonstrate this association, reporting an incidence 2.4- and 5-fold higher, respectively, in men and women, after adjustment for common CVD risk factors (2).

A robust epidemiological evidence has confirmed that HF is among the most common complications of DM. A prevalence of ~20% (4–28%) has been found in clinical trials of glucose-lowering drugs in DM, consistent with a recent position paper of the Heart Failure Association of the European Society of Cardiology (ESC) (3).

DM patients without HF at baseline are more likely to develop this complication over time as compared to non-diabetic people (4), whereas subjects without diabetes at 45 years are more than 60% less likely to manifest HF (5). In the Kaiser Permanente system, out of more than 8,000 patients followed for up to 6 years, the risk of new-onset HF resulted 2.5-fold higher in patients with type 2 DM (T2DM) rather than their non-diabetic counterparts (6). In a large population-based study of 34,198 T2DM patients initially free from overt CVD, HF was even more common than myocardial infarction (MI) as first presentation of CVD (7). In T2DM subjects with newly-recognized HF, the incidence was almost 5-fold higher for HF with preserved ejection fraction (HFpEF) (about 23%) vs. HF with reduced EF (HFrEF) (about 5%) (8).

A low annual incidence of HF (0.2%) and myocardial dysfunction (−0.1%) is reported in type 1 DM (T1DM), likely dependent on the younger age of the studied population (9). Nevertheless, there is a well-documented prevalence of early subclinical cardiomyopathy in children and adolescents with T1DM (10). A meta-analysis of subjects included in clinical trials demonstrated that the presence vs. absence of DM in hypertensive individuals increased the risk of HF by more than 4-folds (11).

Subjects with impaired glucose tolerance (IGT) or insulin resistance (IR) have a 1.7-fold increased risk of HF (12). A community-based cohort study that followed patients for almost 30 years revealed that several biomarkers reflecting IR and dyslipidemia, predicted HF independently of ischemic CVD and other established CV risk factors (13).

HF is a frequent as serious complication of diabetes. Its prognosis is worse than in non-diabetic subjects, with a 75% higher risk of CV death or HF hospitalization (14), and a frequent progression to end-stage HF, which may require heart transplantation despite optimal medical therapy (15). In a prospective study from the mid-1990s, HF 1-year mortality was 30% in people with DM, about 1.5-fold higher than in those without (16). The HF mortality risk was 10-fold higher in a diabetic population older than 65 years (17). Currently, the clinical impact of DM–CMP and other chronic diseases in hospitalized elderly subjects is affected by both gender, and frailty (18–20).

In the CHARM (Candesartan in Heart Failure Assessment of MoRtality and Morbidity) study, DM was associated with a higher relative risk of HF hospitalizations or CV death in patients with HFpEF than HFrEF (21).

On the other hand, as HF is common in DM, so DM is highly prevalent in people with HF, hence one condition increases the incidence and worsens the prognosis of the respective other. Patients with HF have a 4-fold higher prevalence of T2DM (20%) than patients without (4–6%) (22). In a CHARM study group analysis, more than 25% of patients with HF has diabetes (23). When admitted with HF, one-third of patients without a previous diagnosis of diabetes results affected by DM or impaired glucose tolerance (IGT) (24). This prevalence rises to 40% in a large multicenter European study (25), as confirmed in the EVEREST analysis (26).

The mechanism responsible of the increased risk of T2DM in HF is the impaired insulin signaling induced by loss of skeletal muscle mass, sedentary lifestyle, and increased circulating cytokines, which trigger a vicious cycle in which IR and HF deteriorate each other (27). In patients with advanced HF, hemodynamic recovery after ventricular assist device placement is associated with improvements in both systemic and cardiac insulin sensitivity, glucose homeostasis, and toxic lipid products (28). Likewise, IR significantly affects HF prognosis (29).

## The Diabetic Cardiomyopathy

Based on the observation that two-thirds of elderly patients with diabetes presented with a myocardial dysfunction, Lundbæk has firstly suggested in 1954 the concept of a specific DM-related cardiomyopathy (30). This term refers to the current definition proposed by the European Society of Cardiology (ESC), that is a “cardiomyopathy is defined as a heart muscle disease in which the myocardium is structurally and functionally abnormal in the absence of coronary artery disease (CAD) as well as hypertensive, valvular, or congenital heart disorders” (31). Almost 20 years later, Rubler et al. reported the post-mortem findings of four diabetic patients with glomerulosclerosis and advanced symptoms of HF unrelated to valvular, congenital or hypertensive heart disease, alcoholism or significant epicardial coronary artery atherosclerosis. These data thus provided evidence that a cardiomyopathy could directly result from DM, likely in dependence of myocardial microangiopathy or metabolic derangements (1).

Currently, DM-CMP is widely recognized as a specific form of cardiomyopathy which occurs independently of other cardiac risk factors and is promoted by the long-standing metabolic perturbations of diabetes, thus exerting a direct toxic effect on the myocardium (30). Although Rubler originally reported a dilated cardiomyopathy manifesting with the characteristic symptoms of HF, the restrictive LV remodeling with diastolic LV dysfunction is the more frequent picture in DM (32).

In the current clinical practice, DM-CMP diagnosis is still challenging, as it requires the identification of distinct functional and structural changes in the LV and the concomitant exclusion of other cardiac diseases and risk factors for CVD. Due to the very frequent confounding of other HF risk factors such as hypertension, CAD, and renal disease, the burden of a “pure” diabetic cardiomyopathy is conceivable not as high as the cardiomyopathy of heterogeneous etiology, with a calculated prevalence of 16.9% of diabetic patients in a small study (33).

## Pathophysiology of Diabetic Cardiomyopathy

**Figure 1:**
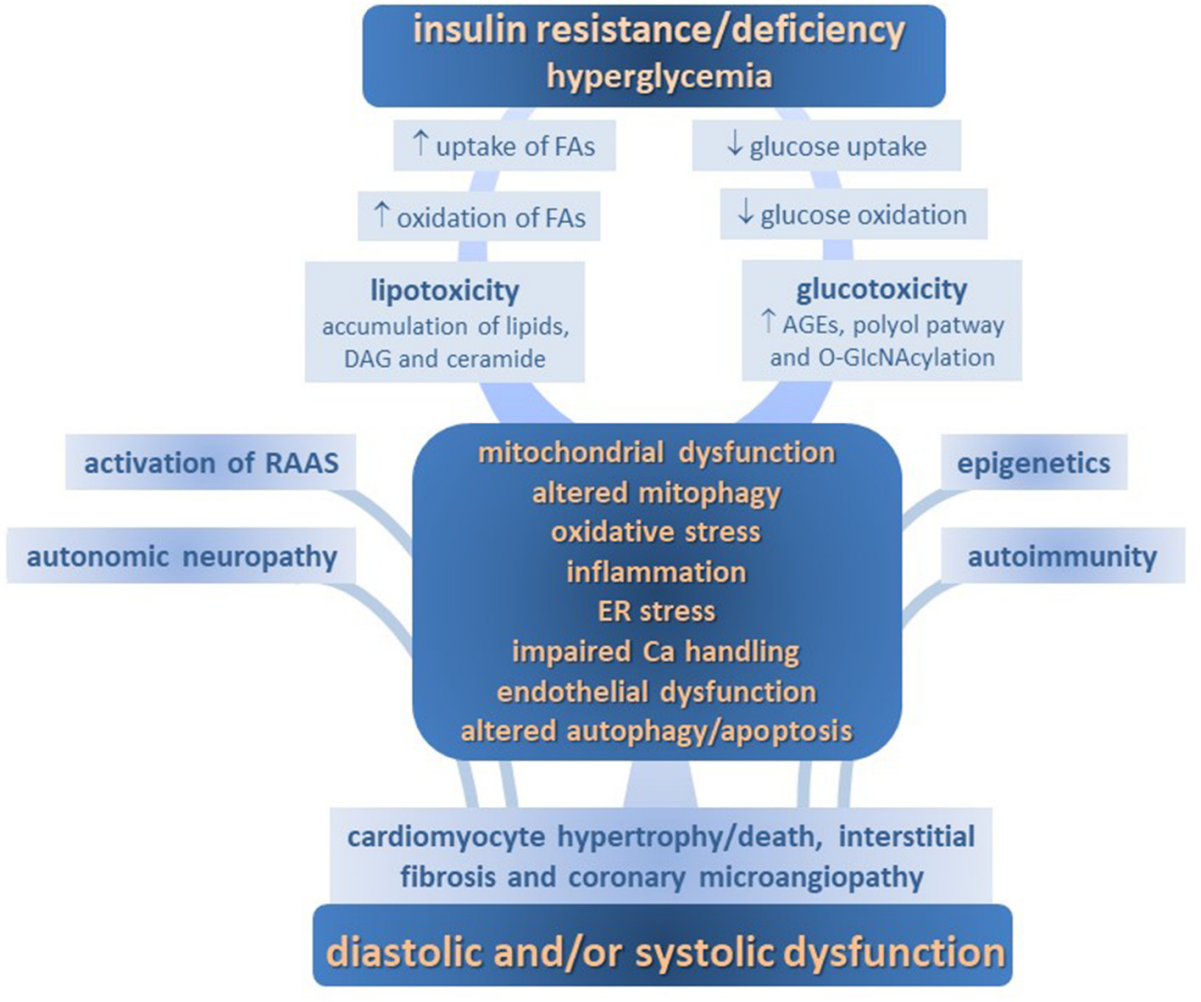
Pathophysiology of diabetic cardiomyopathy.

Several mechanisms determining molecular, cellular and interstitial changes, as well as activation of renin-angiotensin aldosterone axis and adrenergic systems, are involved in the development of DM-CPM. These include imbalance of myocardial energy substrates, gluco- and lipotoxicity, altered insulin signaling, mitochondrial defects, endoplasmic reticulum (ER) stress, deranged intracellular calcium handling, oxidative stress, endothelial dysfunction, deposition of advanced glycation end products (AGEs), maladaptive immune responses, and so on. Each of them contributes to the structural remodeling and functional defects in diabetic myocardium, including impairments in cardiac relaxation, compliance, and contractility (Figure 1).

### Metabolic Abnormalities in the Diabetic Heart

Changes to the metabolic milieu associated with DM, such as lipotoxicity, glucotoxicity and impaired insulin signaling, emerge as crucial pathogenic factors for DM-CMP. Together, they exert, both directly and indirectly, a detrimental increase in oxidative stress, endothelial dysfunction and inflammation, thus making a strong contribution to the myocardium structural and functional derangement.

### Myocardial Energy Substrate Changes and Lipotoxicity

#### Energy Substrates in Healthy Myocardium

Due to the constant cardiac activity, the myocardium is the higher energy-demanding tissue in the body. To absolve this function, it is equipped with an efficient metabolic machinery, mainly represented by the mitochondrial oxidative phosphorylation (34). Under normal conditions, most of energy for myocardium (~60–90%) is supplied by fatty acid (FA) oxidation, whereas the remaining ~10–40% of ATP derives from the oxidation of pyruvate produced in equal amounts by glycolysis and lactate oxidation. Of note, the heart is a net consumer of lactate, both at baseline and upon increase in workload. Ketone bodies are not immediately available from food but produced in the liver by incomplete oxidation of FAs released from the adipose tissue in response either to fasting or energy depletion. They provide, mainly the D-beta-hydroxybutyrate, an alternative substrate for oxidative phosphorylation. Under physiological conditions, aminoacids represent a minor source of energy (21, 35).

The healthy heart is commonly defined a “metabolic omnivore,” due to its crucial capacity to shift between different substrates, according to their availability, in order to ensure a continuous energy supply (36). This metabolic flexibility is mainly determined by the “Randle cycle,” by which high circulating levels of glucose decrease the FA oxidation rates and vice-versa (37). Another metabolic regulator is 5′-AMP-activated protein kinase (AMPK), which acts as a cellular “fuel gauge” (38). In the long term, the nuclear peroxisome proliferator-activated receptor (PPAR)-α, abundantly expressed in the myocardium, play a pivotal role upregulating the transcription of genes related to FA uptake and oxidation (39). Overall, the relative substrate contribution to ATP production can vary mostly depending on energy demand, substrate availability and hormonal milieu. For instance, exercise induces a switch from FAs to glucose oxidation, whereas during prolonged fasting or poorly controlled diabetes, ketone bodies can represent the main energy supplier (34).

#### Changes of Energy Substrates in Failing Heart

In HF the mitochondrial oxidation of FAs is decreased, more likely due to the PPARα signaling suppression and the activation of hypoxia-inducible factor 1α (HIF1α)–PPARγ signaling axis, which impair FA transport into mitochondria and downregulate FA oxidative enzymes (34). Due to the stimulation of lipolysis by sympathetic activation in HF, an increased FA delivery to cardiac myocytes is responsible of this shift away from FA oxidation (40). This imbalance between FA uptake and oxidation leads to cytosolic overload of triglycerides and accumulation of metabolic intermediates generated by non-oxidative pathways such as ceramide and diacylglycerol (DAG), exerting toxic effects and maladaptive signaling, including IR (34, 41). These deposits promote inflammation, cell damage and, eventually, cell death, hence a condition of “lipotoxicity” which contributes to the HF progression (42, 43).

The glucose metabolism of the failing heart is characterized by an enhanced glucose uptake, not accompanied by a concomitant increase in glucose oxidation. Therefore, despite an increase in the relative contribution of glucose oxidation to ATP production, the absolute substrate flux through glucose oxidative pathways actually reduces (34). During the late stages of HF, the glucose availability for ATP production is further impaired by the association of a marked IR (44), even though some authors suggest that the cardiac IR may represent a beneficial mechanism protecting the heart from fuel overload (45).

Ketone bodies may represent a relevant energy source in the HF setting when metabolism of other energy substrates falls (46). Indeed, an upregulation of the enzymes involved in ketone body metabolism is reported in both murine models (47) and patients with advanced HF (48). Since the ATP production/oxygen consumption ratio of the β-hydroxybutyrate is higher (2.50) than that of FA palmitate (2.33), this ketone body has been proposed as a “super fuel” enhancing cardiac metabolic efficiency (34).

#### Metabolic Disturbances in Diabetic Heart

Exposure to hyperglycemia by itself decreases insulin signaling and glucose uptake in cardiomyocytes (49). Essentially, due to the impaired capacity to transport and metabolize glucose determined by insulin deficiency in T1DM and IR in T2DM, the diabetic heart shifts away from glucose as an energy source and gets in a “metabolically inflexible” and less efficient FA-dependent state. This is a crucial pathophysiological condition if considering that glucose is the unique cardiac substrate able to provide ATP during hypoxia or ischemia (50).

Consistent with a prevalent FA utilization, the diabetic heart shows an increased expression of the FA transporter CD36 on both sarcolemmal and endosomal membranes, with an enhanced subcellular vesicular recycling from endosomes to plasma membrane (51). Excess FAs activate PPAR-α, which increases expressions of genes involved in FA oxidation, but also suppresses glucose utilization (52). These typical derangements in the myocardial energy metabolism of diabetic heart are mimicked in mice with cardiac-restricted overexpression of PPAR-α (39). However, studies in diabetic patients either with or without HF argue against an activation of the PPAR-α signaling axis which drives the increase in FA uptake and oxidation (53, 54). Another proposed mechanism for enhanced FA oxidation may be the increased acetylation of mitochondrial β-oxidation enzymes observed in an obese animal model (55).

Excessive FA oxidation increases ATP expenditure for futile cycling of metabolic intermediates, inhibits ATP shuttling from mitochondria to the cytosol, and increases the expression of mitochondrial uncoupling protein (UCP) 3 through PPAR-α, thereby dissipating the mitochondrial proton gradient and deteriorating the ATP production efficiency (56, 57). Finally, these changes produce oxidative stress and mitochondrial dysfunction (58). Moreover, the dissipation of the mitochondrial membrane potential might interfere with excitation–contraction coupling and mitochondrial Ca^2+^ uptake, thus potentially underlying arrhythmias (59).

A deposition of lipids and their metabolites in the cytosol of cardiomyocytes has been documented in DM animal models (60). Human studies using Oil Red-O staining of explanted hearts at the time of heart transplantation have demonstrated cardiac steatosis (28, 41). This excess accumulation of lipids leads to myocardial IR and reduced bioavailability of nitric oxide (NO) (61). Increased levels of DAG in cardiomyocytes activates protein kinase C (PKC) isoforms, thus reducing insulin metabolic signaling and NO production. Similarly, ceramide directly activates atypical PKCs to phosphorylate and inhibit the insulin metabolic Akt signaling and disrupts endothelial NO synthase (NOS) signaling impairing NO bioavailability (62, 63). As well, ceramide may activate caspase 3 and stimulate cytochrome C release, thus inducing cellular apoptosis, and inhibit key pathways involved in defense against DNA damage, such as Poly ADP ribose polymerase (PARP) (64).

A relevant question is whether the altered substrate metabolism is cause or consequence of the failing heart in diabetes. A ventricular biopsy study has showed that even in the absence of contractile failure the diabetic heart exhibits a decreased mitochondrial capacity for β-oxidation, increased accumulation of intracellular lipids, ER stress, and a higher degree of apoptosis (65). Another very recent human bioptic study suggests the crucial role of the toxic metabolic milieu of DM in the early progression of DM-CMP (66). A lipid accumulation in cardiomyocyte was found after only 3 months in non-DM hearts transplanted to diabetic patients. Moreover, triacylglycerol and ceramide contents were both related with early dysfunctions in DM recipients after 12 months. Levels of myocardial insulin receptor were lower in healthy hearts transplanted in DM than non-DM recipients, and SREBP1c (sterol regulatory-element-binding protein-1c) and PPAR systems were highly expressed in cardiomyocytes of DM recipients.

### Hyperglycemia and Glucotoxicity

Sustained exposure to high glucose levels is a major driver of cardiac pathology in DM (67–69). In an observational study on individuals with T1DM, the incidence of HF increased monotonically with the HbA1c, with a range of 1.42–5.20 per 1,000 patient-years between patients in the lowest (<6.5%) and highest (>10.5%) HbA1c categories (70). In a similar study on T2DM patients, each 1% increase in the HbA1c corresponded to an 8% increase in the HF risk (71). Conversely, in T2DM patients of UK Prospective Diabetes Study, each 1% reduction in HbA1c level corresponded to a 16% reduction in the risk of HF (72).

The detrimental effect of chronic hyperglycemia, referred to as “glucotoxicity,” is mainly mediated by oxidative stress, increased formation of AGEs and enhanced substrate flux through alternative metabolic pathways (50).

#### Oxidative Stress

Hyperglycemia contributes to oxidative stress in diabetic heart by excessive oxygen radical formation from the auto-oxidation of glucose, formation of glycated proteins, and impaired buffering capacity due to glycation of metfodant enzymes (73, 74).

The mitochondrial electron transport chain is among the first targets of high glucose levels, with a direct increase in superoxide anion formation. Moreover, high glucose activates protein kinase C (PKC), thus leading to up-regulation of NADPH oxidases (NOX), xanthine oxidase, uncoupling of NO synthase (NOS), microsomal P-450 enzymes, and arachidonic acid metabolism pathways (75). The consequent increased reactive oxygen species (ROS) impair cardiac structure and function by directly damage DNA, proteins and phospholipids, and promote myocytes apoptosis. Kuster et al. found that a short-period of exposure to H_2_O_2_ of *in vitro* rat ventricular myocytes determined a progressive decrease in cell shortening, followed by diastolic arrest. The possible mechanisms were the direct oxidative modification of sarcoplasmic/endoplasmic reticulum calcium-ATPase (SERCA) and Na^+^/Ca^2+^ exchanger (NCX) (76). One harm of the superoxide generation stands in its interaction with NO to form peroxynitrite, a potent oxidant involved in enhanced apoptosis of both animal and human cardiomyocytes (77, 78).

Antioxidant response may be a determinant of the heart health in diabetes. Other findings reveal that the mitochondrial isoform of aldehyde dehydrogenase (ALDH2) may play a role in the development of DM-CMP, possibly through protection against oxidative stress and preservation of mitochondrial integrity (79). Evidence from literature indicates that diabetes upregulates the Ras-related small G protein RhoA, a factor that may impair cardiac function determining uncoupled eNOS, reduced NO bioavailability, and enhanced O${}_{2}^{-}$. IGF-I is a crucial cardiac survival factor that downregulating RhoA produces beneficial effects also mimicked by the Rho kinase inhibitor Y27632 and BH4, a finding indicating that the selective IGF-I overexpression may represent a therapeutic potential for DM-CMP (80).

Enhancing cardiac endogenous antioxidant capacity is an attractive way to prevent DM-CMP. A pivotal target may be represented by Nrf2, an important regulator of cellular detoxification responses and redox status that can lead to antioxidant response elements (ARE)-mediated basal and inducible expression of more than 200 genes (81). Sulforaphane, a molecule within the isothiocyanate group of organosulfur compounds from cruciferous vegetables, such as broccoli, Brussel sprouts or cabbage, is a potent Nrf2 activator (82). A study on *db*/*db* mice fed with broccoli sprout extract or sulforaphane for 3 months showed significant prevention of diabetes-induced cardiac oxidative damage and inflammation by up-regulating Nrf2 transcriptional activity (83). A recent study on mice provided the direct evidence that the preventive effect of sulforaphane against DM-CMP depends on AMPK resulting from both improvement of AMPK-mediated lipid metabolism and potentiation of antioxidative pathway mediated by AMPK/AKT/GSK3β signaling (84).

#### Accumulation of Advanced Glycation End Products

Persistent hyperglycemia causes the non-enzymatic glycosylation of proteins and enzymes with production of toxic AGE adducts, irreversibly altering their structure and functions (85). As an example, AGEs formed on SERCA2a in diabetes impair the sarcoplasmic reticulum (SR) Ca^2+^ reuptake in cardiomyocytes and slow cardiac relaxation (86), whereas long-term treatment with an AGE crosslink breaker partially normalized SR Ca^2+^ signaling (87).

A significant increase in AGE compounds and their binding to cell surface specific receptors (RAGEs) trigger a cascade of pathophysiological responses responsible of severe cardiac damage. Among these, the activation of PKC and NOX lead to the fabrication of peroxide and, ultimately, of ROS, and to the maladaptive activation of mitogen-activated protein kinase (MAPK) and nuclear factor kappa B (NFκB) signaling, followed by the production of several inflammatory and/or profibrotic factors, as well as upregulation of apoptosis (*via* p53 and calcineurin signaling) and autophagy (88–92). All these mechanisms may cause functional and structural damage, till cardiomyocyte death and eccentric LV remodeling with systolic dysfunction.

Interestingly, metformin induces activation and phosphorylation of MAPK, which could mediate its several extraglicemic effects (93, 94).

As shown by light microscopic immune-histochemical visualization, AGEs also accumulate in the myocardial interstitium between cardiomyocytes (95). The non-structural compartment of extracellular matrix (ECM) is represented by a variety of proteins (including collagen IV, laminin, fibronectin, myelin, tubulin, plasminogen activator 1, and fibrinogen), vital for ECM plasticity and with glycosylation as a common denominator (96). Besides ECM disturbation by oxidative stress and inflammation, accumulation of AGEs in the interstitium stimulates the differentiation of fibroblasts into myofibroblasts (*via* Janus kinase-signal transducer and activator of transcription, JAK-STAT signaling), which produce excess matrix proteins, and the crosslink matrix metalloproteinases (MMPs), which indeed impair ECM degeneration. The increased resistance of connective tissue to enzymatic proteolysis and the enhanced collagen cross-linking lead to myocardial fibrosis and stiffness, thus resulting in impaired compliance and diastolic LV relaxation (97–99). This process is potentially mediated by the up-regulation of pro-fibrotic cytokines such as transforming growth factor-β (TGF-β) and connective tissue growth factor (CTGF) (100).

In DM-CMP, an abundant AGEs deposition even involves both endothelial and smooth muscle cells of myocardial microvasculature by triggering vascular inflammation and dampening endothelial NO production (101, 102).

As evidence for the role of AGEs in DM-CMP pathogenesis, the cleavage of preformed AGE crosslinks with ALT-711 attenuates the diabetes-associated cardiac abnormalities in rats (103), and the administration of a RAGE antagonist in a rat model of T1DM prevents AGEs/RAGE signaling-mediated increases in myocardial collagen, fibrosis, stiffness and diastolic dysfunction (104).

The soluble RAGE (sRAGE) is the circulant isoform of RAGE which, by competing with cellular RAGE, may inhibit the pro-inflammatory and pro-fibrotic activity of AGE (105). Unsurprisingly, lower levels of circulating soluble receptors for AGEs predict incident HF in patients with DM (106).

A recent study on experimental diabetes has demonstrated that the inhibition of AGE formation by aminoguanidine exerts a beneficial effect against cardiac remodeling and contractile dysfunction, likely through the regulation of autophagy and ER stress (107).

#### Activation of Polyol Pathway

In In a high-glucose state as diabetes, aldose reductase converts a part of glucose overload to sorbitol, which is oxidized to fructose by sorbitol dehydrogenase. The first reaction produces a depletion of NADPH, a molecule essential for the functioning of various endothelial enzymes, including cytochrome P450 and NO synthase, and a cofactor in the generation of the reduced glutathione. The second reaction increases the cytosolic NADH: NAD^+^ ratio, which can inhibit the glyceraldehyde-3-phosphate dehydrogenase (GAPDH), and increase the concentrations of triose phosphate, with consequent formation of AGE and DAG (108).

The chronic elevation of DAG in diabetes (and in part the increased circulating levels of FAs) activates PKC, a central player in signal transduction and intracellular crosstalk, by phosphorylating a huge array of substrates on serine/threonine residues. PKCβ2 isoform is over-expressed in the myocardium of diabetic animal models and patients with HF (109, 110), and the activation of the PKC/DAG signaling pathway is associated with biochemical and structural changes typical of DM-CMP (e.g., reduced blood flow, increased vascular permeability, basal membrane thickening, ECM deposition, and cardiac hypertrophy) (111–113). On the contrary, PKC inhibition may reverse structural and functional derangements in the diabetic heart (114).

#### Maladaptive Hexosamine Biosynthesis

During chronic hyperglycemia, a small percentage of glucose is shuttled through the hexosamine biosynthesis pathway, thus generating the O-linked β-N-acetylglucosamine (O-GlcNAc). This metabolite may rapidly bind to a multitude of proteins altering their function *via* the O-GlcNAc transferase (115). The ones specifically involved in the progression of DM-CMP include Ca^2+^/calmodulin-dependent protein kinase II (CaMKII), phospholamban and myofilaments, with a negative impact on cardiac contractility and relaxation (116).

Several studies have suggested that O-Glc-N-Acylation of cardiomyocyte proteins might be associated with the development of cardiac hypertrophy (117, 118). This pathogenic mechanism of myocardial hypertrophy has been recently confirmed both in cultured cells and *in vivo*, as triggered by high carbohydrate diets (119). The reduction of the excess cellular O-Glc-N-Acylation, indeed, obtains beneficial effects on calcium handling and diabetic cardiac function (120).

Many mitochondrial proteins are highly susceptible to O-Glc-N-Acylation, which suggests another way for hexosamine pathway to induce cardiac dysfunction in diabetes (121).

### Insulin Resistance

Increasing evidence points to IR as a primary etiologic factor in DM-CMP development.

IR impairs the myocardial glucose utilization and increases the expression of myocardial UCPs. The resulting decline in the efficiency of high-energy phosphate production prevents the myocardial adaptive response to injury, as observed in patients with HFpEF (122, 123).

IR impairs the phosphatidylinositol 3-kinase/protein kinase B (PI3K/Akt) signal transduction pathway to elicit normal metabolic responses. The resultant reduction of glucose oxidation decreases the Ca^2+^ ATPase activity and moves Ca^2+^ back into the SR, thus increasing the intracellular content of ion (30, 124). Since PI3K/AKT can also activate endothelial NOS (125), the reduced NO production in IR states further increases the intracellular Ca^2+^ levels and Ca^2+^ sensitization in cardiomyocytes *via* the cGMP/PKG signaling pathway (30, 51, 126). On the other hand, through the PI3K/Akt pathway, the higher insulin levels associated to IR may induce the titin switching toward the stiff N2B isoform, thus impairing cardiomyocyte distensibility (127).

All these abnormalities may promote cardiac stiffness and diastolic dysfunction, being mainly relevant to restrictive/HFpEF phenotype of DM-CMP, especially in obese T2DM patients. Other contributing mechanisms of IR to myocardial injury are lipotoxicity, sympathetic up-regulation, inflammation, oxidative stress, and fibrosis (128).

The impact of IR on cardiac morphology and function has been extensively documented in clinical studies. In the Framingham Heart Study, LV mass and wall thickness increased with worsening glucose intolerance, and the relation between IR and LV mass observed only in women, was largely dependent on obesity (129). A recent longitudinal study with a 25-yrs follow-up period revealed that cumulative exposure to DM or higher IR adversely affects LV remodeling and function (130). A link between IR and concentric LV remodeling and hypertrophy is confirmed in studies using cardiac magnetic resonance imaging (131, 132).

Intriguingly, opioid system, which seems related to IR (133), play a role in HF (134).

## Pathophysiological Mechanisms Promoting DM-CMP

A plethora of mechanisms mostly connected to the above-described metabolic alterations, act in unison to promote cardiomyocyte injury and cardiac dysfunction in DM.

### Altered Calcium Homeostasis and Calcium/Calmodulin Dependent Protein Kinase II

Perturbations in the cytosolic calcium trafficking and ventricular excitation-contraction coupling at cardiomyocyte level are the mechanistic hallmark of cardiac dysfunction in diabetes (124). Physiologically, the excitation of the cardiomyocyte determines the actin-myosin interaction and contractile activity by inducing Ca^2+^ influx *via* L-type Ca^2+^ channels in the plasma lemma and subsequent Ca^2+^ transient, i.e., Ca^2+^ release from sarcoplasmic reticulum (SR) through ryanodine receptors. During cardiomyocyte relaxation, Ca^2+^ actively moves from cytoplasm into SR by SERCA, with the contribution of sarcolemma Ca^2+^ extrusion by NCX and Ca^2+^ATPase (135).

In diabetic cardiomyocytes, the activity of SERCA and NCX is impaired, likely by either reduction in protein levels or its post-translational modification because of non-enzymatic glycosylation (136). The slower Ca^2+^ transients and leaky Ca^2+^ release channel, result in an impaired calcium load of SR, which is the primary organelle for handling intracellular calcium. To support the correlation between Ca^2+^ handling and cardiac dysfunction, cardiac overexpression of SERCA2a significantly improves myocardial contractility in streptozotocin-induced diabetic rats (137). Being the calcium efflux from cytosol depressed, the cardiomyocyte relaxation impairs, and the action potential duration prolongs (138). These changes are likely associated with the clinical finding of diastolic dysfunction.

CaMKII is a multifunctional serine/threonine kinase physiologically activated in response to β-adrenergic receptor signaling, which targets a number of Ca^2+^ homeostatic proteins in the heart (139). During acute cardiomyocytes activation, CaMKII stimulates glucose uptake, energy production, sarcolemmal ion fluxes, SR Ca^2+^ release/reuptake and myocyte contraction/relaxation coupling, all mechanisms empowering the physiological cardiac adaptation. In diabetic myocardium, as a result of impaired Ca^2+^ handling and oxidative, nitrosative and hyperglycemic stresses, CaMKII is in a state of chronic maladaptive upregulation leading to inefficient substrate utilization, mitochondrial dysfunction, inflammation, fibrosis, ion channel remodeling, impaired intracellular Ca^2+^ handling, contractile dysfunction, and increased risk of arrhythmias (140, 141). In a recent study, the cardiac tissue from both T2DM patients and rats presents an elevated CaMKII activation as compared to non-diabetic controls. Moreover, the trabeculae from diabetic rats have reduced contraction and relaxation performance, which may be restored by the inhibition of this kinase (142).

### Mitochondrial Dysfunction, ER Stress, and Altered Mitophagy

The increased β-oxidation exceeding the respiratory capacity of mitochondria in diabetic hearts induces accumulation of toxic lipid metabolites and generation of oxidative stress and inflammation, which further deteriorate mitochondrial function, possibly culminating in cardiomyocyte death (143). In addition, the signaling pathways by which AMPK activates the PPAR-γ coactivator-1α (PGC-1α), the master metabolic regulator of mitochondrial biogenesis and respiratory function, is impaired in advanced DM-CMP (51).

The hyperglycemia-stimulated ER stress may be the initiator, concomitantly with the FA overload of cardiomyocytes, of an adverse mitochondrial remodeling in human diabetic myocardium (144). ER stress is a condition of over-accumulation of misfolded proteins triggered by intracellular buildup of saturated FA and oxidative stress (145). If the activation of the “unfolded protein response” aiming to restore a normal ER function fails, the cardiomyocyte may go toward a profound mitochondrial dysfunction, including decreased ability to process FA up to self-destruction by apoptosis (146). Upregulation of GRP78 and induction of CHOP, two markers of ER stress response, has been recently described in LV myocardium from diabetic patients (65), consistent with previous findings in animal models of T2DM (147).

Mitophagy, a type of selective autophagy where the damaged or unnecessary mitochondria are sequestered by auto-phagosomes and degraded by lysosomes, is an essential step in maintaining mitochondrial homeostasis in the heart, together with mitochondrial fission, fusion, and biogenesis (148). Increasing lines of evidence suggest that mitophagy is significantly changed in diabetic cardiomyocytes, and some vital proteins involved in this process have been found altered in many diabetic tissues, including heart (149, 150). Even in the context of metabolic syndrome, cardiac mitophagy is altered (151).

### Autophagy, Apoptosis, and Senescence of Myocytes

Adult cardiomyocytes rarely proliferate, thus their death may represent the *primum movens* for the cascade of hypertrophic and fibrotic LV remodeling leading to progressive heart dysfunction, till congestive HF. Higher rates of myocyte death, as determined by autophagy, apoptosis, and senescence, characterize DM-CMP (152).

Constitutive autophagy, a highly conserved process for bulk degradation and recycling of cytoplasmic components in lysosomes, is a homeostatic mechanism crucial to counter oxidative stress and AGE formation and to protect cardiomyocytes from aging-related and ischemia-induced cardiac hypertrophy (153, 154). Bellot et al. reported that ROS and autophagy mutually regulate and that elimination of ROS-damaged cells *via* autophagy is a protective mechanism (155). Indeed, if autophagy is suppressed and excessive ROS persists, the cardiomyocytes would eventually go toward apoptotic death (156). On the other hand, excessive induction of autophagy may indiscriminately destroy cytosol and organelles and determine hypertrophy and fibrosis, with an accelerated progression to ventricular dilatation and decline in systolic performance (157).

The concomitant release of autophagy-related factors, as observed under high-glucose conditions, may contribute to cell death and cardiac dysfunction (158).The activation of PI3K/Akt/mTOR signaling pathway, instead, an essential regulator of cardiac autophagy (159), ameliorates hyperglycemia–induced cardiac hypertrophy (160). A study convincingly supports insulin signaling as a significant regulator of myocardial autophagy, mediating in early life its physiological postnatal suppression, thereby linking nutrient sensing to postnatal cardiac development (161).

Whether the autophagic responses are adaptive or maladaptive remains controversial. Likewise, the role of autophagy in diabetic heart has been not fully understood yet. Several reports show an increased/decreased/unchanged autophagy in the hearts of either humans or animals with T2DM (162). In a study on animal models, autophagic adaptations in DM-CMP seem remarkably different between T1DM and T2DM, being overactivated in the first, but suppressed in the second (163), but even on this topic data are controversial (164). Likely, autophagy regulates both cell survival and cell death in diabetic heart through a strict cross-talk with apoptotic pathways (152), and apoptosis is involved in DM-CMP mainly as a consequence of autophagy dysregulation (165, 166).

A significant increase of apoptosis and cell necrosis characterizes both animal models and patients with DM. Endomyocardial biopsies in diabetic patients with dilated cardiomyopathy show a 4-fold increase of necrosis in cardiomyocytes, 9-fold in endothelial cells, and 6-fold in fibroblasts as compared to their non-diabetic counterparts (167). Hyperglycemia-induced ROS production speeds up apoptosis, some of which is elicited by angiotensin II and glycosylation (168). Many other factors (e.g., mitochondrion damage, oxidative stress, ER stress, inflammation, and even fibrotic signaling) can activate either pro-apoptotic or necrosis signaling pathways in the diabetic heart (169).

The phenomenon of senescence is typically attributed to telomere shortening after repeated cell division. Currently, we know that senescence is also inducible by a series of pathogenic stimuli involved in apoptosis, such genotoxic, mitochondrial and oxidative stresses, as well as inflammation. Moreover, the accumulation of senescent cells can itself cause persistent inflammation and oxidative stress *via* a so called “senescence-associated secretory phenotype” leading to organic dysfunction (169). It is also well-known that senescent cells contribute to the outcome of a variety of cardiac diseases, including age-related and -unrelated cardiac diseases like DM-CMP (170). In this context, DM may impair the *in vitro* proliferation and differentiation potential of adult cardiac stem/progenitor cells, further worsening their senescence phenotype, even when compared to non-diabetic ischemic patients (171).

### Inflammation

Likewise to the known contribution of inflammation to other HF etiologies, both systemic and local maladaptive inflammation responses are strongly concerned with the progression of DM-CMP (172, 173).

Exposure of heart to glucose or FA excess activates NFκB, a protein complex which controls DNA transcription and induces the expression of proinflammatory cytokines (IL6, pro-IL18, pro-IL1β, and TNF-α) and the assembly of NLR family pyrin domain-containing 3 (NLRP3) inflammasome (30, 51). Similarly, AGE/RAGE signaling promotes NF-κB activation and mediates an inflammatory reaction by heterodimerizing with toll-like receptor-4, thus leading to the production of NLRP3, pro-IL1β, and pro-IL18 (104). Activated NLRP3 inflammasome plays a crucial role in the pathogenesis of HF in diabetes, resulting in amplification and infiltration of inflammatory cell, whereas a decrease in NLRP3 attenuates cardiomyopathy in a T2DM rat model (174–176).

Monocytes/macrophages are leading players in DM-CMP pathogenesis. Particularly, macrophage proinflammatory M1 polarization is increased and macrophage M2 anti-inflammatory response inhibited in diabetic heart (177). The recruitment of these cells to sites of inflammation is induced by the C-C chemokine receptor type 2 (CCR2) (178), and macrophages derived from CCR^2+^ monocytes are required for adverse left ventricle remodeling (179). A recent study on mice demonstrated that the heart expression of CCR2 associated to persistent hyperglycemia leads to DM-CMP development, whereas the inhibition of this chemokine could inhibit oxidative stress and M1 macrophage infiltration in diabetic hearts (180).

Apart from macrophages, an involvement of neutrophil and lymphocyte regulation in DM-CMP has emerged. Chronic systemic inflammation in diabetes leads to leukocyte activation and recruitment to various organs with further inflammatory tissue remodeling over time ultimately evolving in fibrosis. At heart level, this may result in reduced cardiac output that ultimately stimulates further cardiac inflammation and fibrosis leading to dilation and established heart failure (181). These pathways may be critical to the discovery of new targeted therapies for controlling DM-CMP progression. As an example, the T cell-specific deletion of sphingosine 1-phosphate receptor 1 (S1PR1), as well as the administration of the S1PR1 antagonist FTY720, are able to exert protection against cardiac fibrosis in a streptozotocin-induced diabetic model (182, 183).

Recently, the role of adipokines (e.g., adiponectin) on the cardiovascular outcome has been well-described (184). Moreover, several less investigated mechanisms might be involved in cardiovascular inflammation (185–187).

### Endothelial Dysfunction

Regardless of the relevance for both accelerated atherogenesis and microvascular diabetic complications, the impaired endothelial function of coronary microvessels is a key feature of DM-CMP (188), especially contributing to diastolic dysfunction and HFpEF (189).

The hallmark of ED is the impaired endothelium-mediated arterial vasodilation as a consequence of depressed bioavailability of nitric oxide (NO), a short-living mediator generated from L-arginine by endothelial NOS (eNOS) (190). During the early stages of IR and DM-CPM, the impaired NO-induced vasodilatation may be balanced by the either preserved or even enhanced endothelium-derived hyperpolarizing factor (EDHF)-mediated vasodilatation. Later, even this mechanism degenerates, thereby promoting microvascular dysfunction (30, 191).

Exposure of endothelial cells to excessive and/or fluctuating blood glucose levels can stimulate the generation of ROS and AGEs, with the consequent downregulation of eNOS and production of NO and cGMP (192, 193). In addition, superoxide anion inactivates NO by forming the more powerful oxidant peroxynitrite, thus triggering nitrosative stress and premature endothelial senescence (188).

The low NO bioavailability to adjacent cardiomyocytes decreases cGMP production and protein kinase G (PKG) activity, with consequent increased ratio of titin isoform N2B:N2BA expression and of intracellular Ca^2+^ content and sensitization. These changes result in a slow relaxation, high diastolic stiffness, and impaired cardiomyocyte elastance (194). As support to the relevance of this mechanism, PKG administration to cardiomyocytes isolated from DM-CMP patients with this phenotype corrects their high resting tension (99). Similar alterations have been observed in cardiomyocytes isolated from patients suffering from both aortic stenosis and DM (195). In addition, ED is associated with microvascular inflammation due to an increased expression of adhesion molecules and local infiltration and accumulation of macrophages expressing TGF-β. As a consequence, myocardial fibroblasts transform into myofibroblasts responsible of interstitial fibrosis (188). The role of TNF-alpha on ED has also been observed (196).

Notably, an increased albuminuria, marker of renal ED, is strictly related to a poor CV outcome in diabetic patients (197–200).

### Microvascular Rarefaction

Similar defects in endothelium-dependent/independent vasodilation involve coronary microcirculation in both T1DM and T2DM patients (201). In addition, structural microvascular alterations impairing the capacity of coronary vascular bed independently of coronary atherosclerosis, may also contribute to DM-CMP (202).

In the myocardium of a well-recognized murine model of diabetes, a significant decline in microvessel density, reduced expression of selected VEGF isoforms, and increase in oxidative stress have been described, all significantly associated with measures of LV performance (203). In a study on patients with end-stage HF, capillary rarefaction and pericyte loss, accompanied by decreased contractility and increased stiffness, characterize diabetic human myocardial explants as compared to non-diabetic samples (204). In the same study, *in vitro* experiments on murine endothelial cells have shown that hyperglycemia attenuates tube formation, migration, and pericyte attraction upon proangiogenic stimulation (204). Moreover, the relative microvascular rarefaction resulting from cardiomyocyte hypertrophy is itself sufficient to induce cardiac fibrosis and diastolic dysfunction (205).

### Autoimmunity

Immune inflammation is involved in the pathogenesis of myocarditis and cardiomyopathy (206). An immune biopathology has also been suggested in the pathogenesis of DM-CMP, especially in autoimmune-prone T1DM patients.

MI has been reported to induce sustained proinflammatory CD4^+^ T-cell and auto-antibody responses against α-cardiac myosin heavy chain, a major autoantigen in myocarditis, both in mice models and in patients with T1DM, but not in control mice and T2DM subjects. Shared cardiac myosin autoantibody signatures between post-MI in T1DM patients and non-diabetic patients with myocarditis also suggests a post-infarction autoimmune syndrome in T1DM patients (207).

Some authors suggested that the cardiac insults of severe diabetic ketoacidosis might initiate the synthesis of antibodies directed to cardiac self-antigens involved in the early immunopathogenesis of cardiomyopathy in young patients with T1DM (208). By measuring prevalence and profiles of cardiac autoantibodies in longitudinal samples of T1DM patients from the Diabetes Control and Complications Trial, poor glycemic control has been demonstrated as associated with cardiac autoimmunity, as shown by the presence of multiple cardiac autoantibody types (209).

### Epigenetics

Epigenetics, the inheritable changes in gene expression without change of DNA sequences, represents a significant link between environmental exposure, as hyperglycemia, inflammation, and oxidative stress, and alterations in gene activity (210).

MicroRNAs (miRNAs) are a group of small, single-strand RNA molecules belonging to the non-coding RNA family, which affect their target genes at a post-transcriptional level by either inhibiting mRNA or degrading protein production (211), whose dysregulated expression is highly implicated in the pathophysiology of DM-CMP.

Some miRNAs abundantly expressed in cardiomyocytes, such as miR-1 and miR-133a, are reduced in T2DM patients (212). In streptozotocin-induced diabetic rats, miR-133a overexpression is able to improve myocardial contractility through the upregulation of tyrosine aminotransferase, a known regulator of norepinephrine production and β-adrenergic receptors (213). Jeyabal et al. found a considerably decreased miR-9 expression in high glucose-cultivated cardiomyocytes and human DM myocardium (214). Downregulation of miR-30c mediates the pro-hypertrophic effects of hyperglycemia in diabetic cardiomyopathy by upregulating Cdc42 and Pak1 genes (215). Li et al. established that miR-30d leads to cardiomyocyte pyroptosis in DM-CMP by direct repression of Foxo3a expression (216). Cardiac-enriched miR-1 and miR-206 are responsive to hyperglycemia and favor the apoptosis of cardiomyocytes through the negative regulation of the heath shock protein 60 (217). Recent evidence demonstrates that miR-208 and miR-499, together with miR-1 and miR-133, might play a role in the differentiation of stem cells into cardiomyocytes (218). A proposed role for miR-208 in diabetic heart disease is the regulation of myosin heavy chain gene expression (219).

Some literature suggests an involvement of exosomes in DM-CMP, the extracellular vesicles containing a variety of biological components, including miRNAs, proteins and lipids, which mediate the intercellular communication (220). The stress induced by hypoxia, inflammation, and hyperglycemia has been reported to increase protein and mRNA content in endothelial cell-derived exosomes, and the exosomes released from diabetic cardiomyocytes could deliver detrimental components able to initiate endothelial cell dysfunction and impair angiogenesis (30). Of note, heat shock protein 20-engineered exosomes exert beneficial effects *via* the modulation of cardiomyocyte exosome secretion with restoration of normal cardiac function under hyperglycemic conditions (221).

Long non-coding RNAs (lncRNAs) are non-protein coding transcripts longer than 200 nucleotides with both nuclear and cytoplasmic location which regulate gene expression through a variety of molecular mechanisms, including the interaction or competition with other RNAs, DNA binding proteins, and specific regulatory DNA sequences (222). Recently, the lncRNA H19 has been found remarkably reduced in a murine model of DM-CMP as a consequence of hyperglycemia, and to regulate cardiomyocyte apoptosis by targeting VDAC1, a mitochondrial porin involved in ATP transport (223).

Histone acetylation is a rapid and dynamic process mainly regulated by histone acetyltransferases (promoting gene transcription) and histone deacetylases (preventing gene transcription), which represent a major epigenetic mechanism whose deregulation may induce the development of several diabetic complications (224). BRD4, a histone acetylated reader protein which regulates either the activation or repression of gene transcription, has been recently identified as a critical mediator of hyperglycemia-induced cardiomyocyte hypertrophy and cardiac fibrosis through the AKT pathway (225).

A study in streptozotocin-induced diabetic rats has recently found that DNA methyltransferase-1 enhances cardiac fibroblast autophagy in diabetic cardiac fibrosis through inhibiting androgen receptor axis (226).

### Activation of the Renin-Angiotensin-Aldosterone System

In a context of IR and hyperglycemia, the inappropriate activation of RAAS despite a state of salt and volume excess, plays an important role in the development of DM-CMP (30), whereas the RAAS block protects against cardiac damage (227).

Beyond receptors AT1 and AT2, Ang-II interacts with NOX, resulting in an overload of oxidants and free radicals in the body, with the subsequent exacerbation of oxidative stress and inflammation (169). This effect is supported by studies showing the effectiveness of ramipril in preventing upregulation of p47phox, p22phox, and reducing NADPH driven oxide production (228). Blocking of Ang-II also reduces the expression of p22phox, NOX and hyperglycemia-induced p47phox (229).

Activation of RAAS may induce systemic and cardiac IR through the mTOR–S6K1 signal transduction pathway (230). Meanwhile, enhanced angiotensin II type 1 receptor and mineralocorticoid receptor signaling in the myocardium enhance the adaptive proinflammatory immune response and inflammation, including increases in leukocyte adhesion, cytokine expression and macrophage infiltration (231).

### Cardiovascular Autonomic Neuropathy

Diabetes is often associated to both neurosensorial damage and neuropathy (232). In particular, diabetic Cardiac Autonomic Neuropathy (CAN), in the absence of cardiac disease, seems associated with LV systolic and mainly diastolic dysfunction, even though it is difficult to assess its independent role among the multitude of factors involved in DM-CMP (233).

Due to an initial predominant parasympathetic denervation, excessive sympathetic activation in the early stages of diabetic CAN may promote LV hypertrophy, thus affecting both sympathovagal balance and baroreflexes (234). Moreover, an abnormal norepinephrine signaling may induce myocardial injury and LV remodeling *via* the cytotoxic effects of the increased catecholamine heart content observed in diabetic rat ventricles (235), eventually mediated by oxidative stress, inflammation, and apoptosis (236–238).

On the other hand, the sympathetic denervation associated to long-lasting diabetic CAN may impair β-adrenergic signaling and reduce myocardial contractile strength, relaxation kinetics, and diastolic distensibility (63, 239).

By changes in myocardial neurotransmitters, CAN may also alter myocardial blood flow and directly deteriorate LV function. A diastolic dysfunction associated to abnormal cardiac sympathetic function appears early in the course of T1DM, as assessed by cardiac sympathetic imaging (240). Among subjects with T2DM or IGT referred for elective coronary angiography, those suffering from CAN have a higher prevalence and a more severe form of LV diastolic dysfunction (241). In a study based on cardiac magnetic resonance imaging in a large cohort of patients with T1DM, the presence of CAN is associated with an increased LV mass and concentric remodeling (242).

## Structural Changes In Diabetic Cardiomyopathy

The above-described detrimental pathways elicited by diabetes at a systemic level and in the myocardium itself collectively promote myocardial hypertrophy and interstitial fibrosis, the two structural hallmarks identified in animal models and patients with both T1DM and T2DM (32, 243). Depending on the combination patterns of these two structural changes, the clinical phenotype of DM-CMP varies from a subclinical diastolic dysfunction to diastolic HFpEF and, eventually, to systolic dysfunction and HFrEF (30, 244). In the HFpEF phenotype, the LV is usually hypertrophied and stiff with normal LV volume. At the cellular level, cardiomyocytes appear hypertrophied with a normal structure of the sarcomere accompanied by increased collagen deposition in the interstitial space. HFrEF phenotype is usually associated with increased LV volume due to dilation, and cardiomyocytes appear damaged, with loss of sarcomeres, and at times replaced by fibrosis (63).

### Cardiac Hypertrophy

Epidemiological data report diabetes and high-sugar diets as risk factors for cardiac hypertrophy and other complications (63, 243–245), a condition highly prevalent (up to 56%) in asymptomatic T2DM patients (246–248). Cardiac hypertrophy is strongly associated with the progression to HF, particularly if hypertension coexists (249), and with a higher incidence of other clinical events, including stroke and sudden death (250).

Cardiac myocytes are differentiated cells which have lost the propensity of proliferation after birth. When exposed to high glucose stress, they increase in size by enhanced protein synthesis and addition of sarcomeres, but not in number, with a resulting greater length (eccentric hypertrophy) or width (concentric hypertrophy) (251). A re-expression of fetal genes has been observed, such as myosin heavy chain (β-MHC) and GATA-1, and activation of early response genes (252).

The microvascular endothelial dysfunction may contribute to the cardiomyocyte enlargement through the parallel addition of sarcomeres due to the removal of a NO-dependent brake on pro-hypertrophic stimuli (188). The increased thickness of ventricular walls in hypertrophied diabetic hearts may partly depend on ECM enlargement. Accordingly, abnormally increased myocardial echodensity, more likely related to collagen deposition, has been detected in asymptomatic diabetic patients with normal ventricular mass (253).

Hypertrophy and fibrosis are two coexisting structural aspects of DM-CMP, likely generated by common pathophysiological mechanisms. As an example, the loss of cardiomyocytes typical of the diabetic heart stimulates the resident cardiomyocytes to compensatively work and become hypertrophic, but at the same time it evocates inflammation pathways generating fibrosis.

### Extracellular Remodeling and Interstitial Fibrosis

**Figure 2:**
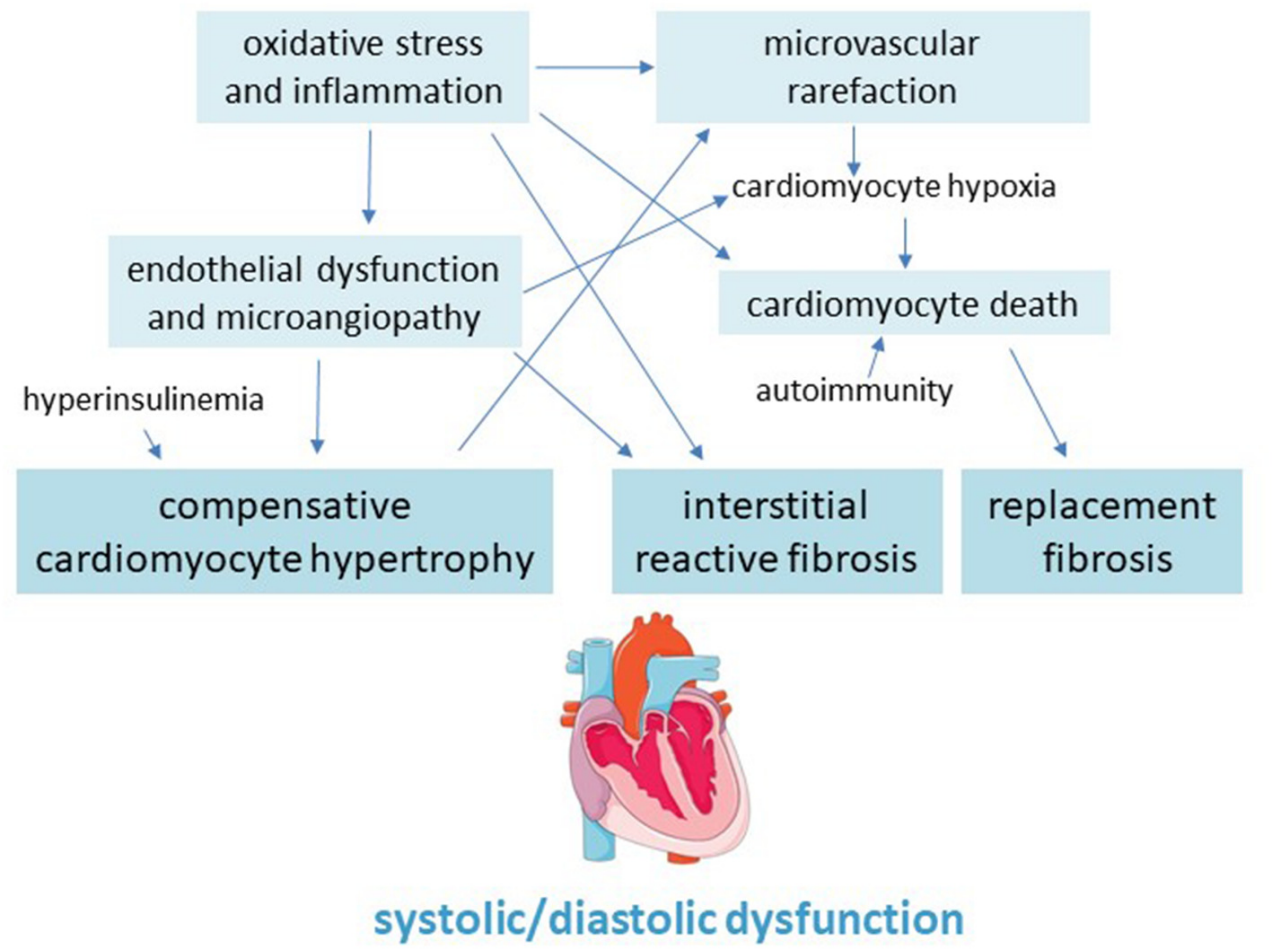
Pathogenic scheme of hypertrophy and fibrosis in the diabetic cardiomyopathy.

Myocardial fibrosis is a main pathological feature of the diabetic heart which involves both left and right ventricular walls, and can lead to cardiac remodeling, dilation and dysfunction, as well as to arrhythmias and, eventually, congestive HF (101) (Figure 2).

Cardiac fibroblasts, the primary matrix-producing cells in the myocardium, help maintaining ECM homeostasis in healthy hearts (254). The majority of resident cardiac fibroblasts responsible for fibrotic response arise from the embryonic epicardium. During development, these cells undergo epithelial-mesenchymal transition under the influence of several growth factors; subsequently, a portion of these mesenchymal cells invade the myocardium to become the resident cardiac fibroblasts. Studies have also revealed that cells of the endocardium, a specialized cardiac endothelial lining, and endothelial cells of the coronary vessel may migrate into the *interstitium* where they undergo endothelial-mesenchymal transition and respond to pro-fibrotic stimuli in a manner similar to resident fibroblasts. Other cells such as pericytes of cardiac vessels can differentiate into collagen-producing cells and may contribute to the fibroblast population following cardiac injury. Finally, the circulating fibrocytes are bone marrow-derived cells considered a potential source of fibroblasts in the fibrotic heart. They represent a unique fibroblast progenitor population that co-express fibroblast markers, along with typical hematopoietic markers (181).

Mechanical or bioactive pathological insults may induce the phenotypic transition of fibroblasts from a resting to an active state characterized by heightened proliferation, migration, contractility, and ECM production (255). A high activation of fibroblasts has been observed in hearts of db/db mice and atrial tissue derived from T2DM patients, resulting in a dynamic balance disorder of cardiac ECM synthesis and accumulation, along with an excessive collagen deposition (256–258).

A dysregulation of specific collagen degrading metalloproteinases (MMPs) and their tissue inhibitors (TIMPs), two crucial determinants of interstitial accumulation of secreted matrix proteins, also contributes to increased extracellular collagen content in the diabetic heart (259, 260). Recently, the enhanced expression of two isoforms of MMP-2 has been induced by high glucose *in vitro* and in a T1DM murine heart model (261).

The pathological processes referring to diabetes which mainly remodel ECM include hyperglycemia, AGE accumulation, inflammation, oxidative stress, and increased levels of neuro-hormones (258).

Excessive collagen deposition may derive from either hyper-expression of TGF-β or CTGF, two key modulators of collagen production. The former is either mediated by angiotensin II activation or induced by high glucose and leptin *via* increasing transcription, secretion, and activation (169, 262, 263). On the other hand, in a study on a murine model of obesity and IR given a diabetogenic diet for 11 weeks, cardiac fibroblasts acquired enhanced myofibroblastic/fibrotic gene expression but reduced responsiveness to TGF-β1 (264). Similarly, cardiac fibroblasts isolated from db/db mice exhibited elevated collagen synthesis but weakened TGF-β1 response (256).

Myocardium tissues of diabetic rats and cardiac fibroblasts treated with high glucose show a significant increased expression of the calcium sensing receptor (CaSR), a member of the C family of the G protein coupling receptor superfamily widely expressed in both prokaryotic and eukaryotic cells (265). A CaSR inhibitor may alleviate the myocardial fibrosis induced by high glucose (266).

The integrins, a family of transmembrane proteins able to integrate and transduce mechanical and biochemical signals, may have a key role in myocardial fibrosis by inducing myofibroblast differentiation (267). Collagen treated with methylglyoxal, a major cell-permeant precursor of AGEs, appears to initiate a forward-feedback loop where glycated ECM increases the expression of integrins. The stiffed myocardial matrix further activates integrins and up-regulates TGF-β, with worsened cardiac fibrosis. Indeed, the deletion of the α11 integrin in streptozotocin-treated diabetic animal models attenuates the cardiac fibrosis (64, 268).

In addition to fibroblasts, even fibrogenic actions by monocytes and macrophages, lymphocytes, endothelial cells and pericytes, mast cells, and cardiomyocytes may contribute to the diabetes-associated heart fibrosis (169).

## Diabetes-Induced Left Ventricular Dysfunction

Even though some authors have postulated that HFpEF and HFrEF represent distinct phenotypes of DM-CMP (63), these clinical patterns are traditionally described as two stages occurring during diabetes progression. An early stage characterized by increased myocardial stiffness, enhanced atrial filling pressure and impaired diastolic function, may be followed, even though not so commonly, by a late stage of further impairment in diastolic function and appearance of a systolic dysfunction (269).

LV diastolic and systolic dysfunctions can be efficiently detected by echocardiography thanks to its large availability and low cost (243). Unfortunately, screening approaches including B-type natriuretic peptide, exercise stress testing, and more sensitive echocardiographic measurements, have not been fully validated yet to identify subclinical dysfunction in diabetic patients (270).

### Diastolic Dysfunction

LV diastolic dysfunction displays from heart stiffening due to both myocardial fibrosis and hypertrophy (100) and represents the initial and most common functional deficit of diabetic heart, generally previous the appearance of systolic dysfunction (271, 272).

An impaired diastolic functioning of LV is detected in 40–75% of asymptomatic T1DM/T2DM patients by conventional echocardiography and tissue Doppler imaging, being characterized by a delayed and extended diastolic phase, with impaired early diastolic filling, prolongation of isovolumetric relaxation, increased atrial filling and increased myocardial stiffness, predominantly in late diastole (249).

Changes in diastolic function have also been widely reported in diabetic animals without evidence of heart disease by other factors (273). In a study of the 90s, diastolic dysfunction has been associated with aging, long duration of diabetes, increased blood pressure, interventricular septal thickness, dyslipidemia, and high HbA1c (274).

### Systolic Dysfunction

As DM-CMP insidiously proceeds and eccentric cardiac remodeling develops, systolic dysfunction may appear, a condition associated with a poor prognosis with an annual mortality of 15–20% and a higher incidence of congestive HF and sudden death (249).

Defects in excitation-contraction coupling at the cardiomyocyte level, including impairment in cardiomyocyte contraction, relaxation, and cytosolic calcium trafficking, as well as epigenetic mechanisms and enhanced mitochondrial ROS generation, may all contribute to this progressive worsening (32, 124).

Even though systolic dysfunction usually follows diastolic dysfunction at a later stage of the DM-CMP course, some studies have detected systolic dysfunction in diabetic patients with normal diastolic function, suggesting that diastolic dysfunction may not necessarily be the first functional alteration (275). In a T2DM population with no documented cardiovascular disease and no signs of ischemia at stress test, asymptomatic LV dysfunction was detected in 262 patients. Among these, 27% had isolated systolic dysfunction and 16% isolated diastolic dysfunction (276).

## Effects of Anti-hyperglycemic Drug Therapy on Heart Failure In Diabetes

Along with the classic outcome of major adverse CV events, recently published CV outcomes trials of anti-hyperglycemic drugs include analysis of HF data, especially the rate of hospitalization for this event.

The ancient drug metformin was absolutely contra-indicated in patients with HF until 2007 when FDA removed this limitation. The controversy regarding its safety and effectiveness in the setting of HF was resolved by the results of a later systematic review of observational studies including 34,000 patients favoring the metformin as the treatment of choice in patients with diabetes and HF (277).

In addition to raised concerns about increased MI, the use of the thiazolidinediones (TZDs) rosiglitazone and pioglitazone, was associated with fluid retention and increased risk of HF, as indicated by three randomized controlled trials, DREAM, ProACTIVE, and GSK211, reporting a respective relative risk of HF of 2.17 (95% CI 0.96–0.91), 1.49 (1.23–0.80), and 7.09 (1.60–0.96) (278). The main mechanisms accounting for TZD-related fluid retention is the PPAR-γ stimulation of EnaC-mediated renal salt absorption in the collecting duct, with the likely contribution of stimulation of sodium transporters in the proximal tubule. Concurrently, the reduction of systemic vascular resistance by TZD might expose the capillary networks to higher perfusion pressures thereby precipitating fluid extravasation. Additionally, TZDs increase the plasma concentration of the VEGF, a potent inducer of vascular permeability, further predisposing patients to oedema (279).

Three new classes of anti-hyperglycemic agents have been introduced in recent years.

The dipeptidyl peptidase-4 (DPP-4) inhibitors exhibited increased HF hospitalization in the SAVOR-TIMI 53 trial evaluating saxagliptin and in the secondary analysis of the EXAMINE trial for alogliptin. A recent pooled analysis illustrates that DPP-4 inhibitors do not increase the HF risk among T2DM patients with a previous history of HF, but they increased this risk among patients without history of HF (HR 1.21, 95% CI 1.04–1.41, *p* = 0.01), possibly because nearly all studied subjects had established CVD (280). Basic research suggests that the inhibition of DPP-4 may exert beneficial actions on heart, mainly by inhibiting the degradation of stromal cell-derive factor-1, a chemokine produced by stromal and endothelial cells that promotes regeneration and repair during organ damage, and that of GLP-1, thus restoring cardiac remodeling and apoptosis caused by the pathological decline in circulating GLP-1 in response to pressure overload (281, 282). On the other hand, since DPP-4 involves in the degradation of vasodilator factors and the NO-dependent mechanism, its inhibition can exert important systemic vasodilator effects that reduce heart load (283). Unfortunately, these beneficial results on animal studies were not replicated in humans.

The antagonists of the GLP-1 receptors (GLP-1RAs) represent the other incretin-based therapy potentiating endogenous GLP-1. Based on the evidence from RCTs, none of the six available GLP-1RAs has displayed benefits against HF, despite demonstration in animal models and humans of ameliorated endothelial dysfunction, improved myocardial function, and cardiomyocyte protection against glucolipotoxicity and ROS (280). A novel GLP-1RA, the oral hypoglycemic peptide 2 (OHP2), has demonstrated to protect against DM-CMP in high-fat diets and continuous streptozocin injection induced rat models. Both hyperlipidemia and myocardium lipid accumulation were decreased by OHP2 treatment. In addition, OHP2 reversed oxidative stress and mitochondrial dysfunction in diabetic hearts (284).

The inhibitors of the sodium glucose co-transporter 2 (SGLT2) are the first class of glucose-lowering agents that have demonstrated in large-scale studies an impressive reduction in the risk of serious new-onset HF events by ≈30% in T2DM patients with or without established CVD (285). Of note, in none of the trials this benefit is explained by the glycemic control. Several mechanisms have been postulated for such a striking cardioprotective effect. The primary action of SGLT2 inhibitors reducing sodium and glucose uptake in the nephron, leads to a decrease in preload and afterload through osmotic diuresis. Additional beneficial effects are improvement of the composition of proinflammatory and anti-inflammatory cytokines in the body, as well as reduction in cardiac fibrosis (286). Other potential cardioprotective mechanism includes the increase in hematocrit, determined by erythropoietin hyperproduction by renal fibroblasts when the stimuli of hyperglycemia and excess glucose reabsorption are removed, the increase in fasting levels of ketone bodies with enhanced utilization of this efficient metabolic fuel in the failing heart, and the inhibition of the sodium hydrogen exchanger-1 in the myocardium, whose overactivity may lead to increase in intracellular sodium and calcium (287).

## Different Aspects of Cardiomyopathy In T1DM and T2DM

Various small and large animal models of T1DM and T2DM have been generated to investigate the impact of diabetes on the heart and a lot of clinical studies have been published in the last decades on DM-CMP. Nonetheless, the complex pathophysiology of this condition remains still less than fully clear. The topic is further complicated by the different etiology of T1DM and T2DM that make partially distinct the mechanisms involved in their cardiac dysfunction (288).

Although etiologically different, the two types of diabetes share common metabolic disturbances, including hyperglycemia, dyslipidemia and associated glucotoxicity, lipotoxicity, and oxidative stress that are the predominant pathological mechanisms driving the development of DM-CMP as determined by insulin deficiency in T1DM and insulin resistance in T2DM.

On the other side, the development of HF in T1DM appears more closely related to glycemic control than in T2DM as indicated by the reported different increase in HF risk, 30% in T1DM and 8% in T2DM patients, for each additional percentage point of HbA1c (270). Likely, a good metabolic control obtained by insulin therapy in patients with T1DM may normalizes the metabolic derangements induced by insulin deficiency and attenuate the detrimental effects of diabetes on the heart (288). Instead, the insulin resistance typical of T2DM leads to increase in circulating triacylglycerol levels and FA delivery to cardiomyocytes that result in impaired mitochondrial β-oxidation, with greater mitochondrial dysfunction and accumulation of toxic lipid metabolites in the heart of patients with T2DM than in patients with T1DM (30).

Differences in pathophysiology of heart damage between the two types of diabetes also result in different clinical pictures. In T2DM-associated DM-CMP, there is a prevalence of mechanisms mediating concentric LV remodeling and hypertrophy with increase in ventricular stiffness leading to diastolic dysfunction. The corresponding clinical features include reduced ventricular compliance with increased systemic and pulmonary venous pressures and congestion despite preserved systolic function. By contrast, T1DM-associated diabetic cardiomyopathy is characterized by cardiomyocyte loss, LV remodeling and increased myocardial collagen deposition, which increase LV end-diastolic volume and impair systolic function. As a consequence, symptoms of systolic dysfunction are more typical in patients with T1DM with earlier clinical manifestations of HFrEF (289).

A similar progression of DM-CMP has also emerged in preclinical studies in diabetic animal models. A study comparing cardiac performance in rat models of T1DM (streptozotocin induced) and T2DM (Zucker diabetic fatty rats) by a pressure-volume conductance catheter system, suggested that a decreased systolic performance and a delayed relaxation mainly characterize T1DM, whereas an increase in diastolic stiffness of the heart is more remarkably in T2DM (290). A recent study using speckle-tracking echocardiography with invasive hemodynamics for the detection of cardiac dysfunction in rat models of T1DM and T2DM confirmed these results (291). It was found that contractility and active relaxation were deteriorated to a greater extent in T1DM compared to T2DM. In contrast, diastolic stiffness was more pronounced in T2DM. Correspondingly, systolic function was markedly altered in T1DM but preserved in T2DM, a disease profile resembling that observed in T2DM patients with HFpEF.

## Conclusion

Diabetic cardiomyopathy is a common complication of diabetes which deserves a special clinical attention due to its insidious subclinical progression that, in some cases, may culminate in a manifest and rapidly evolving HF burdened by a very poor outcome.

The main driving force of the pathological processes specific of DM-CMP is hyperglycemia, a factor centrally placed among multiple interwoven pathways involving complex cellular and molecular perturbations which affect both myocardial structure and function.

Despite the current large knowledge, the pathophysiology of DM-CMP development and progression is still far from being fully elucidated. Consequently, effective therapies targeting this diabetic complication are lacking.

In-depth knowledge of etiologic and pathogenic mechanisms is crucial for the development of target-specific treatments to reduce the risk of HF in diabetic patients. Since subclinical cardiac abnormalities could be reversible when early detected, prevention-oriented therapies can even hopefully be identified.

## Author Contributions

TS and FS: conceptualization. TS, PP, RG, GA, AD, AC, EV, LR, and FS: investigation. TS: writing—original draft preparation. TS, FS, and PP: writing—review and editing. TS, FS, and RG: supervision. All authors have read and agreed to the published version of the manuscript.

## Conflict of Interest

The authors declare that the research was conducted in the absence of any commercial or financial relationships that could be construed as a potential conflict of interest.

## Abbreviations

AGE: advanced glycation and product
AMPK: 5′-AMP-activated protein kinase
CaSR: calcium sensing receptor
CaMKII: Ca^2+^/calmodulin-dependent protein kinase II
CAN: cardiovascular autonomic neuropathy
CCR2: C-C chemokine receptor type 2
cGMP/PKG, cyclic guanosine 3′: 5′-monophosphate/ protein kinase G
CTGF: connective tissue growth factor
CV: cardiovascular
CVD: cardiovascular disease
DAG: diacylglycerol
DM: diabetes mellitus
DM-CMP: diabetic cardiomyopathy
ECM: extracellular matrix
ED: endothelial dysfunction
eNOS: endothelial NO synthase
ER: endoplasmic reticulum
HF: heart failure
HFpEF: heart failure with preserved ejection fraction
HFrEF: heart failure with reduced ejection fraction
HIF1α: hypoxia-inducible factor 1α
IGT: impaired glucose tolerance
FA: fatty acid
LV: left ventricle
FDA: Food and drug administration
GAPDH: glyceraldehyde-3-phosphate dehydrogenase
lncRNAs: long non-coding RNAs
MI: myocardial infarction
MAPK: mitogen-activated protein kinase
miRNAs: MicroRNAs
MMPs: matrix metalloproteinases
NCX: Na^+^/Ca^2+^ exchanger
NFkB: nuclear factor kappa B
NO: nitric oxide
NOX: NADPH oxidase
NRLP3: NLR family pyrin domain-containing 3
O-GlcNAc: O-linked β-N-acetylglucosamine
PARP: poly ADP ribose polymerase
PGC-1α: PPAR-γ coactivator-1α
PI3K/Akt: phosphatidylinositol 3-kinase/protein kinase B
PKC: protein kinase C
PKG: protein kinase G
PPAR: peroxisome proliferator-activated receptor
RAAS: renin angiotensin aldosterone system
RAGEs: receptor for advanced glycation end products
ROS: reactive oxygen species
SERCA: sarcoplasmic/endoplasmic reticulum calcium-ATPase
SGLT2: sodium glucose co-transporter 2
sRAGE: soluble RAGE
S1PR1: sphingosine 1-phosphate receptor 1
SR: sarcoplasmic reticulum
SREBP-1c: sterol regulatory-element-binding protein-1c
T1DM: Type 1 diabetes mellitus
T2DM: type 2 diabetes mellitus
TGF- β: transforming growth factor-β
TIMPs: tissue inhibitors MMPs
TZDs: thiazolidinediones
UCP: uncoupling protein
VEGF: vascular endothelial growth factor.
